# The effect of celery *(Apium graveolens)* on reproductive parameters in male wistar rat

**Published:** 2016

**Authors:** Wesam Kooti, Najmeh Kafash-Farkhad, Ali Ghorbani Ranjbary, Naim Sharafi-Ahvazi

**Affiliations:** 1 *Student Research Committee, Kurdistan University of Medical Sciences, Sanandaj, Iran*; 2 *Department of Biology, Urmia University, Urmia, Iran*; 3 *Department of Biotechnology, School of Veterinary Medicine, Ferdowsi University of Mashhad, Mashhad,Iran*

**Dear editor**

In recent years, the number of scientific research papers of Iranian scientists has substantially grown in national and international journals that indicates particular attitude of Iranian scientific community to the development of knowledge in different fields. Moreover, improvement of quality of scientific papers is necessary. For this purpose, criticism of published studies is a way to increase the quality of articles and make them clear. In Avicenna Journal of Phytomedicine, volume (5), issue (2), year 2015, an article entitled “Effects of aqueous extract of celery (*Apium graveolens* L.) leaves on spermatogenesis in healthy male rats” was published and the papers like this should be appreciated. However, the paper has some drawbacks which if not resolve, could be misleading for researchers who tend to use it or do research in its direction. So, with all due respect to the research team, we decided to evaluate the paper ambiguities in order to improve the quality of future articles.

## Introduction

In the introduction of this paper, Kerishchi et al. study has been noted which stated “celery has a protective role in the testes and amplifies the sperm parameters“. This report is inconsistent with the author’s report, because Kerishchi et al. reported that celery at some doses has no effect on sperm parameters and pituitary-gonadal axis (Kerishchi et al., 2011 ▶)

Regarding the extraction procedure, just plant systematic approval in another university by unknown person was mentioned. Authors are requested to confirm the plant systematic (also scientific name and species) by agriculture herbarium or Pharmacognosy Department of their University and announce the voucher specimen number. Thus, future researchers will rely more confidently on the project results.

In this study, there is no control group and sham group mistakenly considered as a control group. These two groups are different from each other and it should be considered when used. Interestingly, authors have cited an article of Mokhtari and Zanboori that has separate control and sham groups (Mokhtari and Zanboori, 2011 ▶).

The point of debate in this study is 30 days of animal’s treatment. Authors first declared that “Spermatogenesis is a period of 48 days but most of the changes occur within the first 35 days”. It is noteworthy that authors should have provided complete and accurate results and do not follow “most of the changes”. However, they did not observe the 35- day period and they took only 30 days of treatment into account. Also, they have mentioned another paper in which the investigators treated rats before occurrence of toxicity for up to 23 days (Hamza and Amin, 2007 ▶). In the study of Hamza and Amin, authors discuss poisoning and prevention not the effect of the extract on testicular function.

It should be noted that the temperature in which this study has been done (22±2 ˚C), and generally in authentic articles the period of wistar rats spermatogenesis has been mentioned 52 days, which violates the entire duration of this study ( Freitas et al., 2002 ▶, Momen et al., 2012 ▶, Kooti et al., 2014 ▶). 

Afzalzadeh et al. used a magnification of 400X for sperm count but were not able to see motile sperm. So, how could the authors of this study achieved correct results by a magnification of 100X? On the other hand, the article of Afzalzadeh et al. that was cited for its sperm counts did not use a standard scientific formulas with reliable reference (Afzalzadeh et al., 2013 ▶).

The authors had to mention if both testicles were examined or only one of them (the left or the right). Also, for testis weight it should be mentioned that it is for the left or right testicle. 

Normally, the sperm count is not acheived through photos and only sperm concentration is visible but the latter is lacking here. However, in normal testis, sertoli cells are countable and visible but are not visible in the photo from the control group of this study. Also, image magnification of histological sections results was mentioned to be 300X while in method’s section and also for pathological images a magnification of 100X is used.

The number of spermatozoid in control group which was reported in the result’s section is half of normal Wistar rat spermatozoid in the control group of valid articles and with more precision we noticed that the number of spermatozoid in the experimental groups of this study is equal to the spermatozoid control group of other research (Jalali and Hasanzadeh, 2013 ▶; Kooti et al., 2014 ▶; Dorostghoal et al., 2013 ▶). This contradiction may be due to defect in treatment duration that was previously mentioned and in fact increase in the number of sperm in the experimental groups in this study could not be reliable.

As regards without measurement of pituitary-gonadal axis hormones the boosting effect of plant on spermatogenesis could not be confirmed explicitly (Dorostghoal et al., 2013 ▶; Ghorbani Ranjbary et al., 2014 ▶) and also the results of this study groups, the treatment method and duration, and even images that were provided by the authors are contradictory. So, the data from this report which concluded that celery increases fertility in male rats cannot be scientifically correct. The authors claim about celery positive effects on male fertility needs to be reviewed and is not scientifically verifiable. Also, several studies demonstrated that celery and its compounds has anti-fertility effects (Ohlsson et al., 2010 ▶; Kooti et al., 2014 ▶; Li et al., 2010 ▶; Ghasemiboroon et al., 2014 ▶) which contravene the results of this study and the writers did not mention that in discussion section. All items listed have quite subtle influence on the research results and should be considered if used in the future.
