# Supplementary material for: The effect of celery (Apium graveolens) on reproductive parameters in male wistar rat
Source: Avicenna J Phytomed. 2016 May-Jun;6(3):260–5. (PMC4930532)
Supplement: Related texts

#  Authors reply: 

Hereby the authors of the article under the title “Effects of aqueous extract of celery (*Apium graveolens* L.) leaves on spermatogenesis in healthy male rats” answered the researchers’ questions:

1-In the introduction of this paper, Kerishchi et al. ▶, study has been noted which stated “celery has a protective role in the testes and amplifies the sperm parameters. This report is inconsistent with the author’s report, because Kerishchi et al. reported that celery at some doses has no effect on sperm parameters and pituitary-gonadal axis (Kerishchi et al., 2011 ▶)

Answer: The article of Kerishchi et al. (2011) was carried out using the extract of celery seeds, but our research was done using the extract of celery leaves and since seed and leaf have different compounds, this disparity in results may be justified by the different compounds of seed and leaf.

2- Regarding the extraction procedure, just plant systematic approval in another university by unknown person was mentioned. Authors are requested to confirm the plant systematics (also scientific name and species) by agriculture herbarium or Pharmacognosy Department of their University and announce the voucher specimen number. Thus, future researchers will rely more confidently on the project results.

Answer: In this manuscript, we mentioned “Celery (*Apium graveolens* L.)” which bears the same meaning all over the paper. However, the herbarium number of *Apium graveolens* L. kept in Shahid Chamran University of Ahvaz is 854125. 

3- In this study, there is no normal control and sham group mistakenly considered as a control group. These two groups are different from each other and it should be considered when used. Interestingly, authors have cited an article Mokhtari and Zanboori that has separate control and sham groups (Mokhtari and Zanboori, 2011 ▶).

Answer: In the articles with one control group it is usually called “control”. Also the cited reference (Mokhtari and Zanboori, 2011 ▶) just used it for applying the distilled water not for control or sham group. 

4- The point of debate in this study is 30 days of animal’s treatment. Authors first declared that “Spermatogenesis is a period of 48 days but most of the changes occur within the first 35 days”. It is noteworthy that authors should have provided complete and accurate results and do not follow “most of the changes”. However, they did not observe the 35- day period and they took only 30 days of treatment into account. Also, they have mentioned another paper in which the investigators treated rats before occurrence of toxicity for up to 23 days (Hamza and Amin, 2007 ▶). In the study of Hamza and Amin, authors discuss poisoning and prevention not the effect of the extract on testicular function.

Answer: According to the pathology images and the results mentioned in this paper, we realized that significant changes have been made in the testes; therefore, the period of the present study (30 days) was suitable for the celery extract effects on the spermatogenesis.

5- It should be noted that the temperature in which this study has been done (22±2 ˚C), and generally in authentic articles the period of wistar rats spermatogenesis has been mentioned 52 days, which violates the entire duration of this study ( Freitas et al., 2002 ▶, Momen et al., 2012 ▶, Kooti et al., 2014 ▶). 

Answer: This temperature (22±2 ˚C) was suitable and no adverse effect was shown on the testes in the article that was cited (Ghasemiboroon et al., 2014 ▶). Also, it was used for all animals in the control and treatment groups. 

6- Afzalzadeh et al. 2013 ▶ used a magnification of 400X for sperm count but were not able to see motile sperm. So, how could the authors of this study achieved correct results by a magnification of 100X? On the other hand, the article of Afzalzadeh et al. that was cited for its sperm counts did not use a standard scientific formulas with reliable reference.

Answer: In the study of Afzalzadeh et al. there was probably no motile sperm to see and it was not related to magnification. On the other hand, in the present study the standard method was used for sperm counts. 

7- The authors had to mention if both testicles were examined or only one of them (the left or the right). Also, for testis weight it should be mentioned that it is for the left or right testicle.

Answer: In this article, we used the left testicles, but the results were similar for both of them.

8- Normally, the sperm count is not acheived through photos and only sperm concentration is visible but the latter is lacking here. However, in normal testis, sertoli cells are countable and visible but are not visible in the photo from the control group of this study. Also, image magnification of histological sections results was mentioned to be 300X while in method’s section and also for pathological images a magnification of 100X is used.

Answer: In this article, we used histological images with a magnification of 300X to show significantly different clarity in the treatment groups compared with control group.

9- The number of spermatozoid in control group which was reported in the result’s section is half of normal Wistar rat spermatozoid in the control group of valid articles and with more precision we noticed that the number of spermatozoid in the experimental groups of this study is equal to the spermatozoid control group of other research (Jalali and Hasanzadeh, 2013 ▶; Kooti et al., 2014 ▶; Dorostghoal et al., 2013 ▶). This contradiction may be due to defect in treatment duration that was previously mentioned and in fact increase in the number of sperm in the experimental groups in this study could not be reliable.

Answer: The spermatogenesis in the wistar rats has seven phases and the different results in this manuscript with other articles may be due to this issue.

 10- As regards without measurement of pituitary-gonadal axis hormones the boosting effect of plant on spermatogenesis could not be confirmed explicitly (Dorostghoal et al., 2013 ▶; Ghorbani Ranjbary et al., 2014 ▶) and also the results of this study groups, the treatment method and duration, and even images that were provided by the authors are contradictory. So, the data from this report which concluded that celery increases fertility in male rats cannot be scientifically correct. The authors claim about celery positive effects on male fertility needs to be reviewed and is not scientifically verifiable. Also, several studies demonstrated that celery and its compounds has anti-fertility effects (Ohlsson et al., 2010 ▶; Kooti et al., 2014 ▶; Li et al., 2010 ▶; Ghasemiboroon et al., 2014 ▶) which contravene the results of this study and the writers did not mention that in discussion section. All items listed have quite subtle influence on the research results and should be considered if used in the future.

Answer: In several articles (e.g. Ghasemiboroon et al. (2014) ▶), the authors have said that extract of celery leaves have no adverse effect on the important spermatogenesis hormones (FSH and testosterone) in the male rats; So, the results of the present study were compatible with the previously published articles. 

## References

[B2-1] Afzalzadeh MR, Papahn AA, Amirzargar A, Kazemi Varnamkhasti M, Ganjali H, Gharib Mombeni E (2013). Effect Of Vitis Vinifera Leave Hydro-Alcoholic Extract On Reproductive Parameters In Adult Normal Male Rats. J Phys Pharm Adv.

[B2-2] Dorostghoal M, Seyyednejad SM, Khajehpour L, Jabari A (2013). Effects of Fumaria parviflora leaves extract on reproductive parameters in adult male rats. Iran J Reprod Med.

[B2-3] Freitas F, Cordeiro-Mori F, Sasso-Cerri E, Lucas S, Miraglia S (2002). Al¬terations of spermatogenesis in etoposide-treated rats: a stereo¬logical study. Interciencia.

[B2-4] Ghasemiboroon M, Ghafourian Boroujerdnia M, Ahangarpoor A, Kooti W, Hasanzadeh Noohi Z, Noori Ahmad Abadi M (2014). The effect of hydro-alcoholic extract of celery (Apium graveolens) leaves on serum level of testosterone, FSH and LH in male rats. ZUMS J.

[B2-5] Ghorbani Ranjbary A, Ghorbani Ranjbary N, Ghorbani Ranjbary Z, Jouibar F (2014). Effects of intraperitoneal injection of extracts of origanum vulgare on gonadotropin and testosterone hormones in male Wistar rats. J Babol Univ Med Sci.

[B2-6] Hamza AA, Amin A (2007). Apium graveolens modulates sodium valproate‐induced reproductive toxicity in rats. J Exp Zool A Ecol Genet Physiol.

[B2-7] Kerishchi P, Nasri S, Amin G, Tabibian M The effects of Apium graveolens extract on sperm parameters and H-G hormonal axis in mice.

[B2-8] Kooti W, Ghasemiboroon M, Ahangarpoor A, Hardani A, Amirzargar A, Asadi-Samani M (2014). The effect of hydro-alcoholic extract of celery on male rats in fertility control and sex ratio of rat offspring. J Babol Univ Med Sci.

[B2-9] Kooti W, Mansouri E, Ghasemiboroon M, Harizi M, Ashtary-Larky D, Afrisham R (2014). The Effects of hydroalcoholic extract of apium graveolens leaf on the number of sexual cells and testicular structure in rat. Jundishapur J Nat Pharm Prod.

[B2-10] Li H, Li HB, Zhang M, Yan F, Zhang ZX, Li ZL (2010). Effect of apigenin on the reproductive system in male mice. Health.

[B2-11] Mokhtari M, Zanboori M (2011). The Effects of Lead Acetate on Sexual Behavior and the Level of Testosterone in Adult Male Rats. Int J Fertil Steril.

[B2-12] Momen HR, Eskandari N (2012). Effect of vitamin E on sperm param¬eters and DNA integrity in sodium arsenite-treated rats. Iran J Reprod Med.

[B2-13] Ohlsson A, Ulleras E, Cedergreen N, Oskarsson A (2010). Mixture effects of dietary flavonoids on steroid hormone synthesis in the human adrenocortical H295R cell line. Food Chem Toxicol.

[B2-14] Shalizar Jalali A, Hasanzadeh SH (2013). Crataegus monogyna fruit aqueous extract as a protective agent against doxorubicin-induced reproductive toxicity in male rats. Avicenna J Phytomed.
